# Lipid-Based Nano-Sized Cargos as a Promising Strategy in Bone Complications: A Review

**DOI:** 10.3390/nano12071146

**Published:** 2022-03-30

**Authors:** Supandeep Singh Hallan, Jhaleh Amirian, Agnese Brangule, Dace Bandere

**Affiliations:** 1 Department of Pharmaceutical Chemistry, Riga Stradins University, Dzirciema 16, LV-1007 Riga, Latvia; supandeepshallan@gmail.com (S.S.H.); jalehamirian@gmail.com (J.A.); 2 Baltic Biomaterials Centre of Excellence, Headquarters at Riga Technical University, Kalku Street 1, LV-1658 Riga, Latvia

**Keywords:** solid lipid nanoparticles, nanostructured lipid carriers, exosomes, liposomes, bio-conjugation, bone targeting, bone regeneration, calcium phosphate, bisphosphonates

## Abstract

Bone metastasis has been considered the fatal phase of cancers, which remains incurable and to be a challenge due to the non-availability of the ideal treatment strategy. Unlike bone cancer, bone metastasis involves the spreading of the tumor cells to the bones from different origins. Bone metastasis generally originates from breast and prostate cancers. The possibility of bone metastasis is highly attributable to its physiological milieu susceptible to tumor growth. The treatment of bone-related diseases has multiple complications, including bone breakage, reduced quality of life, spinal cord or nerve compression, and pain. However, anticancer active agents have failed to maintain desired therapeutic concentrations at the target site; hence, uptake of the drug takes place at a non-target site responsible for the toxicity at the cellular level. Interestingly, lipid-based drug delivery systems have become the center of interest for researchers, thanks to their biocompatible and bio-mimetic nature. These systems possess a great potential to improve precise bone targeting without affecting healthy tissues. The lipid nano-sized systems are not only limited to delivering active agents but also genes/peptide sequences/siRNA, bisphosphonates, etc. Additionally, lipid coating of inorganic nanomaterials such as calcium phosphate is an effective approach against uncontrollable rapid precipitation resulting in reduced colloidal stability and dispersity. This review summarizes the numerous aspects, including development, design, possible applications, challenges, and future perspective of lipid nano-transporters, namely liposomes, exosomes, solid lipid nanoparticles (SLN), nanostructured lipid carriers (NLC), and lipid nanoparticulate gels to treat bone metastasis and induce bone regeneration. Additionally, the economic suitability of these systems has been discussed and different alternatives have been discussed. All in all, through this review we will try to understand how far nanomedicine is from clinical and industrial applications in bone metastasis.

## 1. Introduction

Cancer is a major public health issue that reduces the average survival rate of sufferers by 5 years [1]. It can be of various categories including bone, skin, brain, liver cancer. Among them, bone cancer has been considered one of the most prone sites to metastasis after lung and liver due to its physiological supportive environment for tumor development [2,3]. Bone metastasis associated with prostate cancers occurs largely in Europe and America comes second after lung cancer in terms of mortality [4,5].

The bone metastasis takes place in the lumbar vertebrae, thoracic vertebrae, cervical vertebrae, and sacrum parts of the bone, which is not often curable [6]. Bone metastasis occurs when cancer cells distribute themselves from the site of formation to distantly localized skeletal tissues, and hence, speed up the tumor process. Moreover, red bone marrow allows the entry of cancer cells owing to the anatomy of the skeletal system, where they induce innumerable angiogenic and bone resorption factors [7,8]. As a whole, the tumor in bones is the result of bone crosstalk by means of chemokines, and some other soluble proteins [9]. Therefore, unlike bone cancer, which originates from the bones, bone metastasis occurs via cells that break away from different origins. Thus, bone metastasis takes place via hematogenous dispersal [10]. The dispersed tumor cells facilitate the bone remodeling for the better growth of the tumor cells in the bone marrow. Further, these cancer cells not only initiate osteoclast-mediated bone resorption but also the production of growth factors and the secretion of osteolytic cytokines. This whole event ultimately leads to the formation of osteoblastic lesions in patients with prostate cancers [6]. 

Concerning the pathophysiology of bone metastasis, the mechanism of production of osteoblasts (build the bone) and osteoclasts (eat up the bone) is well controlled in a balanced way, known as bone homeostasis. The disproportion in both may result in the shifting of the normal niches into metastatic niches [11]. Metastatic cancer cells in the bone interact with osteoclasts, which play significant roles in early bone colonization [12]. The application of anti-cancer drugs, bisphosphonates, radiopharmaceuticals, etc., can be used to cure bone metastasis. However, the non-availability of the ideal treatment strategy is a major concern due to multiple obstacles including bone breakage, reduced quality of life, spinal cord or nerve compression, and pain [13,14]. 

Nanomedicine is used as both a diagnostic tool and as a drug carrier to the specifically targeted sites. A variety of benefits of nanotechnology can be derived from treating chronic human diseases by delivering the active molecules to specific sites of action. In this regard, plenty of nano-scale transporters are exploited as nanorobots, nanosensors, and actuation materials in living cells [15]. Among them, lipid-based nanomaterials have gained great attention worldwide in the research community due to having certain advantages including high biocompatibility, the ability to entrap both hydrophilic as well as lipophilic active molecules, controlled release, as a non-toxic degradation product, to enhance the stability of entrapped molecules, etc. [16,17]. Moreover, they provide a possibility to design them with surface decorating ligands and different charge-inducing agents [18]. The lipid nanoformulations meant for drug delivery in cancers have attained remarkable clinical and commercial success (Doxil^®^, a liposome formulation encapsulating DOX) and many are still under the clinical trial phase [19,20,21]. 

The aim of the review is to shed light on state of art innovations taking place in the field of bone targeting by means of synthetic or naturally derived lipid-based nano-sized carriers. The numerous aspects, including development, design, possible applications, challenges, and future perspective of lipid nano-transporters, namely liposomes, exosomes, SLN, NLC, lipid nanoparticulate gels to treat bone metastasis and induce bone regeneration will be discussed in a detail. In addition, the economical aspects and challenges in scaling up the lipid nanoformulations will be discussed. The methodology of the review of the literature was carried out using databases Scopus, PubMed, and Web of Sciences to search the publications from the year 2000–2022. Our primary search terms were “bone metastasis”; “bone remolding”; “bio-conjugation in bone targeting”; “liposomes”, “lipid-based drug delivery systems”, “calcium phosphate” etc.

## 2. Strategies in Bone Targeting

**Figure 1:**
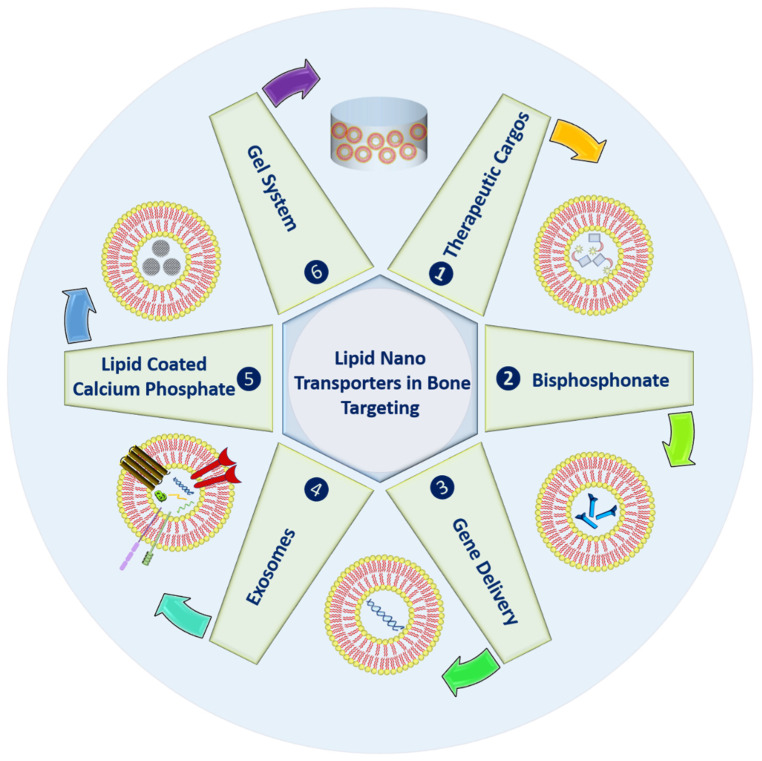
Types of the lipid nano-transporters in bone targeting.

In order to enhance the life of bones, various strategies using lipid-based nanocarriers have been highlighted in Figure 1. These systems can be used to deliver active molecules to the bones for different purposes; they can undergo bisphosphonate conjugation to enhance bone affinity. 

Further, lipid carriers can also provide successful gene knockdown due to well know transfection properties. Naturally derived exosomes or extracellular vesicles can be used for various objectives with enhanced shelf life and prolonged circulation. Inorganic materials, namely calcium phosphate, can also be embedded within lipid carriers for bone remolding with improved bone affinity. Finally, to enhance the residence at the bone site, lipid carriers can be gellified. The above-highlighted possibilities have been discussed in detail in the following sections.

### 2.1. Therapeutic Cargoes

It is very difficult to design a drug delivery system for bone targeting due to the complexity of the solid tumor and the presence of various biological barricades. Some other contributing factors such as hypoxia, pH value of the cell, high interstitial-fluid pressure, physicochemical characteristics of the active drugs, for example, poor water solubility and pharmacokinetic profile, and instability in the harsh physiological environment [22,23,24].

Furthermore, the physical and chemical limitations of the drug molecules that create hindrance in bone targeting can be solved by applying nanotechnology. For example, liposomes can solve the issues of low water solubility and bioavailability, and avoid first-pass metabolism. Similarly, lipid nanoparticles can enhance bone penetration of the drugs, and prevent the drug from rapid oxidation. Apart from this, zero-order release can be achieved by minimizing the dose frequency. Some of these issues have been addressed and solved, limitations of active molecules and their solutions have been given in Table 1.

The foremost hindrance in designing a drug transporter is the burst release of the payload before reaching the targeted site [31]. Undoubtedly, these lipid-based delivery systems have many advantages; still, the biggest challenge is the successful loading of drugs without wasting any fraction and keeping them intact [32]. The right selection of the type of drug carrier with the best fit composition can keep the entrapped drug molecule stable for more than 6 months, which otherwise may deteriorate in 30 days when available in solution form [33].

### 2.2. Bisphosphonate Delivery

Bisphosphonates are non-hydrolyzable pyrophosphate analogous known to interfere with growth and cell signaling by inhibiting the mevalonate pathway [34]. Alendronate sodium (second-generation bisphosphonate) has been categorized under nitrogen-containing bisphosphonate. It has a very high affinity toward bones, which can reduce bone turnover. Therefore, it can be used as a targeting moiety in bone metastatic treatment strategies [35]. 

The presence of bisphosphonates, namely alendronate and zoledronic acid in the drug delivery system can be beneficial in order to attain an improved pharmacokinetic profile of anticancer molecules. These systems can also exhibit sustained high plasma concentration along with slow clearance. In this way, their passive accumulation will ultimately provide a large retention time within the solid tumors [36,37].

**Figure 2:**
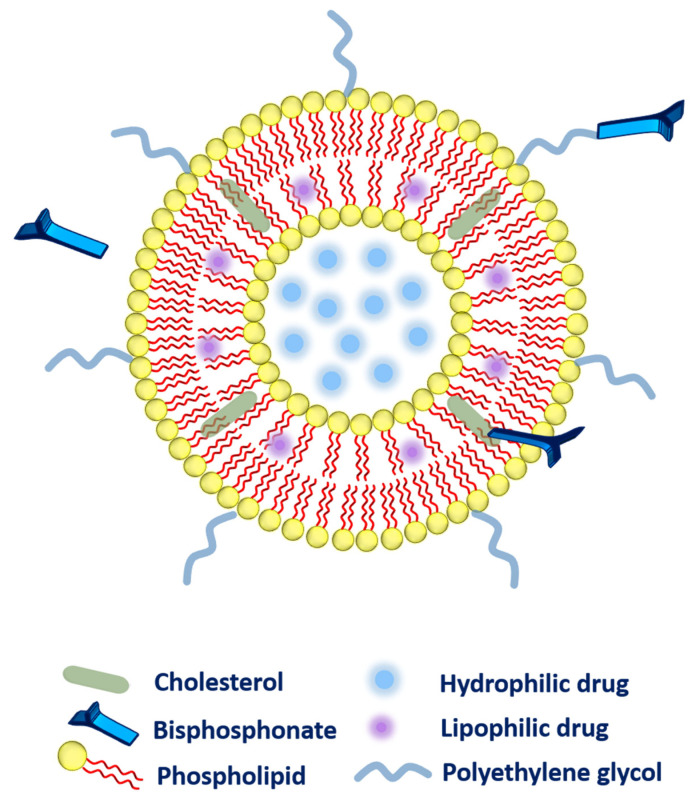
Different possibilities for the association of bisphosphonates with the liposomes.

The method of choice to attach the different bisphosphonates to the liposomes or other lipid-based carriers has been conflicting. Several methodologies that have been adopted to link the bisphosphonates to the liposomal system has been highlighted as follow (Figure 2).

➢ Firstly, during liposomal production, bisphosphonate (alendronate sodium trihydrate, or zoledronic acid) can be added passively to the aqueous phase of the liposomes owing to high water-solubility [37,38,39].
➢ Secondly, the bisphosphonate can also be conjugated along with the cholesterol (as a main component of liposomal composition via a click reaction known as Cu(I)-catalyzed Huisgen 1,3-dipolar cycloaddition). The aforementioned system has exhibited a very strong affinity toward bones [40].
➢ Thirdly, bisphosphonate can be associated with the polyethylene glycol (PEG) chain to provide higher circulation time to the liposomes. More precisely, phospholipid-PEG-bisphosphonate conjugation can be employed in liposomes [41]. Polyethylene-glycol-conjugated phospholipid was used to embed zoledronic acid (as a potent inhibitor of farnesyl-pyrophosphate synthase). The fabricated liposomes were subjected to biodistribution studies that evidenced higher drug accumulation in the liver, spleen, bones, and tumor as compared to zoledronic acid in free form or entrapped in non-PEGylated liposomes. However, toxicity was the main concern as these liposomes were found very toxic to rodents [36].

Moving ahead, the choice of suitable bisphosphonate is another challenge. Researchers have tried to establish a comparison between different bisphosphonates where liposomal systems composing alendronate have been investigated in T-cell immunotherapy, which delayed ovarian tumor growth in mice. Wherein, a better synergism was established in alendronate and γδ T cells as compared to zoledronic acid. In addition, concerning safety, alendronate has been found safer than zoledronic acid as concluded from cell viability studies. In this manner, the Nitrogen-containing bisphosphonates conjugation has improved the pharmacokinetic profile and enhanced passive retention at tumor sites [38].

Apart from the liposomes, the solid lipid nanoparticles are also flexible enough to deliver bisphosphonates. One study reported wherein, bisphosphonate has been modified along with the surfactant of choice, which facilitated the transportation of the active molecule outstandingly. It is a well-proven fact that the surfactant is the most crucial component of lipid nanoparticles. Interestingly, Brij 78 (a non-ionic surfactant) has been conjugated with pamidronate (bisphosphonate) in order to enhance the overall bone affinity of the lipid-nanoparticles [42]. 

### 2.3. Gene Delivery

Undoubtedly, bone repair is a natural process. Nevertheless, in most cases, it is not always possible due to the inability of the body itself. In this regard, bone morphogenetic proteins (BMP) play a key role in bone regeneration via regulating cartilage and bone differentiation [43,44,45,46]. Therefore, the exogenous delivery of these types of growth factors can speed up the repairing process. Nonetheless, there are so many limitations to the delivery of BMP and liposomes can be a good candidate to encapsulate BMP either by their direct addition or transfection through gene carrying. Until now, no study has been found where these two methods have been compared with each other [47]. Protein delivery faces shortcomings as compared to gene delivery due to major obstacles including high dose, correct folding, and glycosylation of the protein [48].

In this regard, gene therapy is an approach having the capacity to produce intra-cellular proteins and can express them for a longer period along with the regulation of transgene expression. Moreover, gene therapy avoids the use of a higher concentration of therapeutic moieties; hence, can be delivered only one time with a minimal dose. Henceforth, adverse effects associated with a high amount of therapeutics can be avoided. Therefore, combinational therapy of different genes in association with biomaterials could be the most favorable strategy [48,49]. 

Comparing viral gene therapy to non-viral gene therapy is relatively toxic with unpredictable immune responses to the host. Considerably, liposomes with calcium phosphate as a non-viral siRNA vector in gene transfection have been exploited, in which dual functioning is designed firstly to avoid prompt growing of the calcium phosphate particles, secondly to prevent degradation of the entrapped siRNA of the Bcl-2 gene. This proposed system has been designed to deliver the Bcl-2 gene. To make the system pH-triggered charge-reversible, citraconic as a derivative of a maleic amide has been conjugated with cholesterol-aminocaproic acid to coat calcium phosphate particles and the further surface has been tuned by depositing the siRNA. In the last, the positively charged lipid 1,2-dioleoyl-3-trimethylammonium-propanechloridesalt (DOTAP) and negatively charged lipid dioleoylphosphatydicacid (DOPA) has been added and the charge reversal was investigated with the reversal of zeta potential results, which successfully suppress the Bcl-2 without inducing any toxicity in lung cancer A549 cells [50]. In some cases, the carrier should be negatively charged to achieve efficient transfection whilst positively charged in an acidic endolysosome environment to inhibit its uptake by disrupting the endolysosomal membrane. To tackle this difficulty, the charge reversal phenomenon has been applied [51,52].

Apart from this, the conjugation between PEG-alendronate accomplished in the stem cells approach has been considered an effective tool in the treatment of various disorders related to bone degeneration due to its osteogenic potential [53,54]. In this regard, poor bone marrow homing at damaged tissues in response to injury is the main obstacle in the systemic infusion of mesenchymal stem cells (MSC), which have been addressed in a study, wherein the peptide sequence with a strong affinity to bone marrow-derived MSC has been encapsulated into liposomal nanoparticles modified with alendronate sodium (high bone mineral affinity). The aforementioned liposomes were composed of a DSPE-PEG-Alendronate complex for better bone targeting (gene delivery to osteoblastic cells). Further, the biophotonic imaging study assured the successful accumulation of genes in osseous tissue [41]. 

### 2.4. Exosomes 

**Figure 3:**
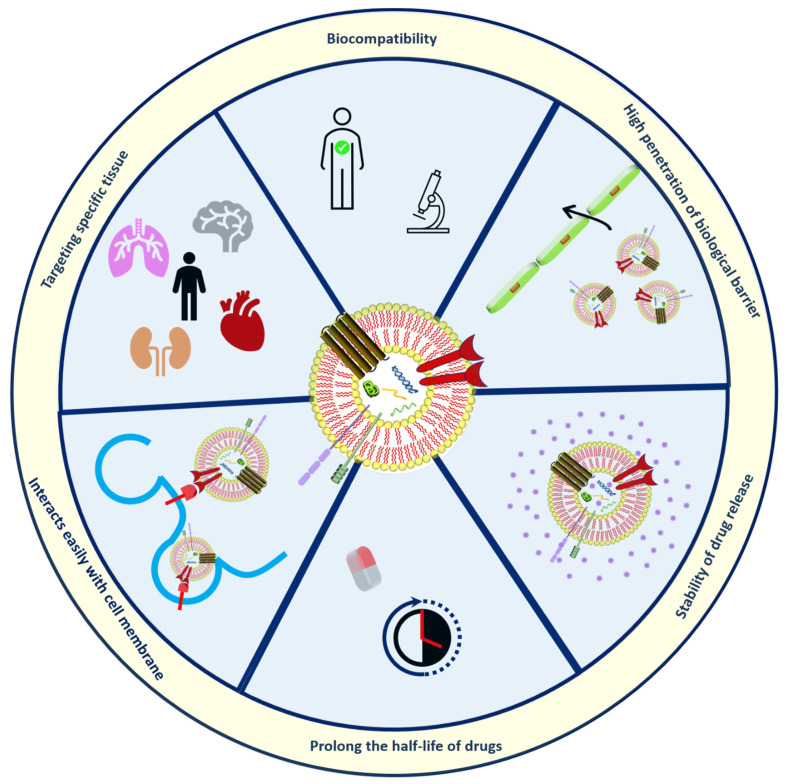
Structural organization and Merits of exosomes.

Exosomes (30–150 nm in diameter) as bio-inspired lipid-based novel systems innately originated from biological sources such as endosomal compartments of animal and plant cells utilized as natural transporters between cells by encapsulating nucleic acids, anti-cancer agents, proteins, genetic lipids [55,56]. The use of exosomes has advantages over artificially prepared nanoparticles discussed in Figure 3.

**Figure 4:**
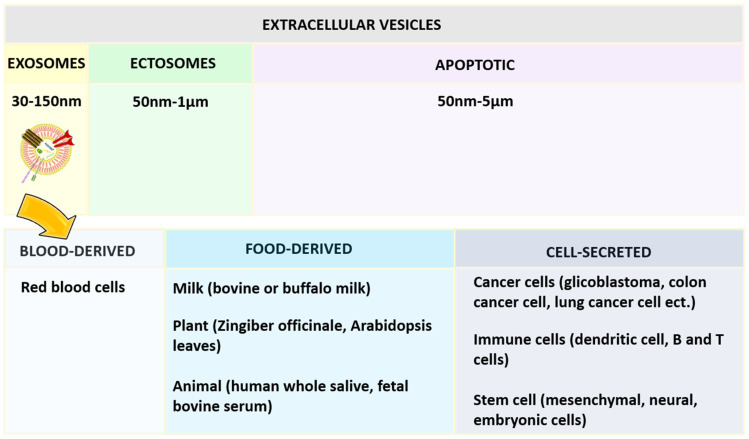
Classification of extracellular vesical based on the diameter and exosomes based on the source of origin.

Exosomes are more reliable because of their ability to communicate in the cells naturally [57]. Based on the diameter, extracellular vesicles can be categorized as shown in Figure 4, namely exosomes, ectosomes, and apoptotic bodies. Among the exosomes, ectosomes, and apoptotic bodies, the exosomes that are most stable can retain the content enclosed for a longer period and are also useful to enhance the bioavailability of active compounds. They can reach desired sites of action by avoiding various digestive or bio-fluid enzymes. One report has concluded that curcumin was four times more stable by loading into exosomes derived from EL-4 cells [58,59].

Exosomes are surrounded by the single external lamela, originating largely from blood, food, and the cell (Figure 4) and widely distributed in plasma, urine, semen, saliva, bronchial fluid, breast milk, serum, amniotic fluid, synovial fluid, tears, lymph, bile, and gastric acid [60]. In the beginning, exosomes were considered waste material from cell damage and cell homeostasis, later they were proposed to carry therapeutic moieties [61]. 

Fascinatingly, the exosomes can disclose the biological information of the eukaryotic cells from where they have been collected practically, which is useful in curing a wide range of diseases such as chronic inflammation, specifically tumors [62,63]. 

Exosomes are developed from the inward folding of the membrane of the early endosomes (originating from the plasma membrane of the cells), later giving rise to multivesicular bodies and taking part in various endocytic and trafficking tasks [60,64]. Further, the high cholesterol fraction containing multivesicular bodies will be guided toward fusion along with the plasma membrane to release exosomes that otherwise undergo destruction by lysosomes. Therefore, this whole event depends on the amount of cholesterol content present in multivesicular bodies [60,65]. Exosomes possess a very complex architecture containing 4400 proteins, 194 lipids, 1639 mRNAs, and 764 miRNAs, which depicts their functional diversity [66,67]. 

Attributable to the complexity of the exosomes based on the structural organization (huge number of proteins), no robust strategy is available to provide their reproducibility. Another possible reason is the variation of exosomes derived from different matrixes. Therefore, a set of different techniques need to be applied, which increases the overall production cost. These are the frictions in the smooth production of exosomes that escalate their use at the clinical level. In general, ultracentrifugation is used for all types of exosomes collections while other methods are also available based on size, immunoaffinity capture, and precipitation of exosomes. Still, these methods are not sufficient enough for the purification of exosomal yield because the result is usually a mixture of non-differentiated content of exosomes and different extracellular vesicles [60]. 

Moreover, in the future new strategies may be evolved involving immunoaffinity and microdevices along with good outputs and higher purified collection. Stremersch, S and co-authors have compared not only the different methods of loading to exosomes (either during biogenesis or after isolation of exosomes) but also to synthetic liposomes for the fusion capacity. No remarkable difference has been noticed in the uptake of all the above-mentioned systems. This study proposed that anionic liposomes were able to target gene knockdown under the same experimental conditions; however, exosomes cannot because of inappropriate/no release of encapsulated miRNA [68].

Liposomes and exosomes are closely related in terms of composing one lipid bilayer, a diameter below 200 nm, and the capability to load both hydrophilic and lipophilic active moieties. In the case of discrepancy, exosomes offered a more complex surface with high specificity due to the existence of numerous proteins (such as tetraspanins). In a typical composition of liposomes, no proteins are associated with the surface of the external membrane. These proteins make possible the efficient targeting and uptake hence, have very good immunocompatibility [69]. 

Although, exosomes face some disadvantages including rapid clearance, low loading capacity, and the non-availability of scale-up manufacturing techniques. In contrast, liposomes provide the possibility for PEGylation to achieve long circulation, well-defined production even at an industrial scale (many liposomal products are already on market), and the feasibility of the ligand-guided approach [69,70]. 

Despite this fact, it has been always a topic of conflict that liposomes are better than exosomes comparatively and vice versa. To neutralize the above statement, the lipid component of liposomes can be used in exosomes to achieve higher intelligence concerning membrane fusion [71,72]. Therefore, the functioning of liposomes and exosomes merging into one system has been investigated. Herein, the surface membrane protein of exosomes hybridized not only along with the functional lipids of liposomes but also PEGylation did enhance the overall colloidal stability of the system. These two systems can be combined through processes namely freeze–thaw, incubation, and sonication [73]. 

Bone-derived exosomes in bone-related complications can play a key role in the regulation of gene expression, migration, and proliferation. In prostate cancer, affected cells secrete both osteoblast and osteoclast stimulating factors that initiate either bone resorption or bone formation, or both. The bone metastasis disturbs the balance between the events of bone-resorbing osteoclasts and bone-forming osteoblasts [74]. In general, bone metastasis-related to prostate cells is osteoblastic in radiographs and exosomal microRNAs derived from the prostate cancerous cells are believed to promote osteoblastic bone metastasis [75,76]. Osteolytic lesions have been traced in all the subjects affected with the osteoblastic metastasis [77]. To understand this mechanism, the role of exosomes derived from osteoblastic, osteoclastic, or combination of human prostate cancer cells have been identified in one study. Wherein, investigators have found that exosomes promoted bone tumor growth via osteoclastogenesis in vitro and induced osteolysis in vivo. MicroRNA delivered through exosomes has inhibited osteoblastogenesis evidenced by inhibition of type I collagen expression in vivo. Hence, aggressive growth of prostate cancer cells inhibited, deteriorated bone matrix, and induced premetastatic niche for tumor growth, thus, playing a key role in bone homeostasis [75].

Exosomes can also be used in bone regeneration using exosome-loaded scaffolds. The incorporation of the exosomes into the scaffolds is completed via physical adsorption (incubation of scaffolds in exosomes solution) [40,78] and in situ gelation via cross-linking [79].

### 2.5. Lipid Coated Calcium Phosphate

Over recent years, lipid-coated calcium-phosphate (LCP) nanoparticles have often been used as a platform to overcome various drug delivery barriers for the delivery of various therapeutics, including gene, protein/peptide, chemotherapeutics, and theranostic agents [80,81,82,83,84,85]. Calcium phosphate (CaP) as a biomaterial ideally resembles the bone matrix, possesses a strong bonding affinity towards bones, and is highly biocompatible. These types of materials are best known for their utility in local delivery into bones [86,87]. The CaP also has been investigated as a dental pulp tissue engineering biomaterial, which makes it suitable in dentistry applications as well [88]. The biominerals having calcium and phosphates are widely present in teeth and exoskeletal structures of invertebrates in ample fractions [89,90]. Significantly, the amorphous calcium phosphate has drawn the huge attention of researchers worldwide. This material is available in a huge variety of compositions in a natural or synthetic form [91]. Because of its large surface area, modifiable degradation rate, this is used as a filling material in dental cavities or chewing gums in order to avoid the demineralization of teeth [92]. 

However, there are certain challenges in its applications, including uncontrollable rapid precipitation leading to poor colloidal stability and dispersity, which hence persuades fluctuation in drug release handling and storage [93,94]. This rapid growth of calcium phosphate-nucleic acids particles circumscribes the in vivo repeatability [95]. These forenamed issues can be avoided by providing a suitable coating to the system and turning the particles into nanoscale [95,96]. Hyaluronic acid, as a possibility in order to enhance the stability of amorphous calcium phosphate, has been investigated. The electrostatic complex formed between the anionic carboxyl group and cationic Ca^2+^ of hyaluronic acid and amorphous calcium phosphate, respectively, was explored [97,98,99]. Further, sodium hyaluronic acid- amorphous calcium phosphate -hexametaphosphate complex has managed to show the stability of dispersion in terms of uniform dispersity for up to 3 months, hence the agglomeration problem has been solved. Moreover, curcumin was loaded to the aforementioned system with 78% encapsulation efficiency successfully achieved controlled release at 30 h at pH 7.4. The system has good biocompatibility and anti-cancer activity assessed on A549 cells [94]. 

Interestingly, in a study reported recently, calcium phosphate has been adopted to coat lipid nanoparticles (first time claimed by the authors) for the treatment of bone diseases namely osteoporosis, osteoarthritis, and bone cancer. The calcium phosphate nano-coating can help in the osseointegration with the bones in vivo. Lipid nanoparticles have been prepared by the cold microemulsion dilution method. The surface of lipid nanoparticles was made charge-activated using negatively charged fatty acid or cationic stearyl amine. Two different methods (positive or negatively charged lipid nanoparticles differently) were adopted for the calcium phosphate coating. The coated surface turns to a higher uptake as compared to non-coated evidenced by their selectivity and promising bone targeting potential and no cytotoxicity has been observed on the application of the calcium phosphate layer. Further, in order to evaluate the dye uptake in human osteosarcoma cells, Sudan Red III as a dye has been loaded, which was available successfully in the bone cells [100].

**Figure 5:**
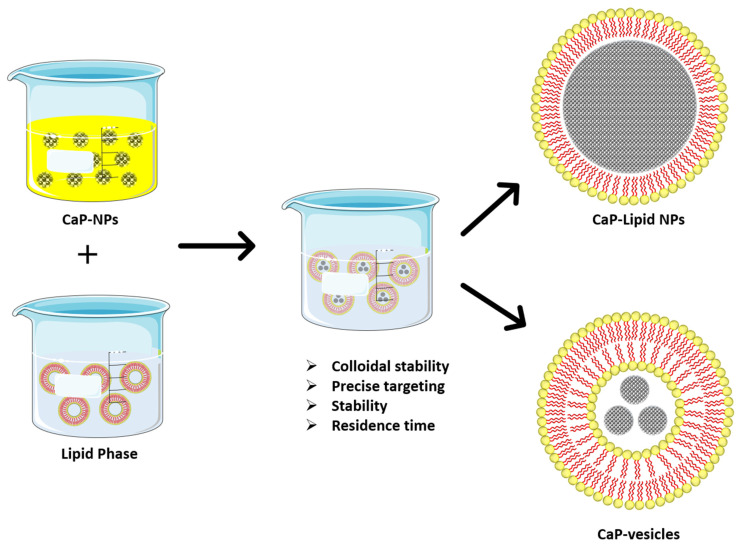
Lipid coating of calcium phosphate nanoparticles namely lipid nanoparticles and/or vesicles are either naturally derived (exosomes) or artificially (liposomes).

Further, lipid-coated systems have become the center of interest for researchers, thanks to their biocompatible and bio-mimetic nature [101]. The use of lipid coating has been represented in Figure 5. Likewise, another case study has been performed turning the calcium phosphate into a lipid system as a potential cargo in dental regeneration applications. Inflammation of dental pulp (Pulpitis) is the main reason toothache occurs due to overexpressed proinflammatory cytokines and innate immune response. The pulp inside the tooth comprises nerves, blood supply veins, vascular and connective tissues. This condition shortens the tooth life and it is highly challenging to find the best suitable strategy to avoid damage to the pulp tissues [88,102]. PEGylated liposomes have been fabricated and loaded with calcium phosphate as a successful suppressor of the inflammatory cascade (tested by quantitative polymerase chain reaction) and encouraged the proliferation of dental pulp stem cells [88]. 

The calcium phosphate as a solid core has been chosen to carry high-density lipoprotein, namely Apolipoprotein E3 categorized under the fat-binding proteins family. The designed system has been enabled to cross the blood–brain barrier and performed targeting and apoptosis of glioblastoma cells. A dual objective has been pointed out in this study wherein siRNA was entrapped into CaP solid core and decorated by Apolipoprotein to make an efficient siRNA delivery at the cytoplasmic level. Taken together, efficient delivery of Apoprotein and lipid-coated CaP-siRNA-nanoparticles successfully targeted Ras-activated brain cancer cells, and have undergone macropinocytosis in a Ras-dependent mechanism [103]. 

Moving ahead, the research has been proposed by Cai et al. [50], wherein calcium phosphate–lipid hybrid nanoparticles are associated with the charge reversal property were synthesized for gene silencing purposes in lung cancer. SiRNA Bcl-2 gene via phosphate–lipid hybrid nanoparticles successfully reached the cytoplasm and expression of Bcl-2 in A549 cells. 

### 2.6. Gel System 

Even though bone tissues have regeneration potential, the reformation of bones is still challenging at the clinical level. The application of the lipid nanosystems in bone regeneration is very hard due to the short retention time at the bone defect site upon injection [104]. In this regard, the use of hydrogel in bone regeneration in the form of engineered scaffolds and nanomedicine is expected to be a promising strategy not only at in vivo and in vitro levels but also at the clinical level in the future [105]. 

Hydrogels are mainly 3D cross-linked hydrophilic polymers/homopolymer/copolymer with hydro-swellable property and can be implanted in bones in various shapes and sizes [106]. 

**Figure 6:**
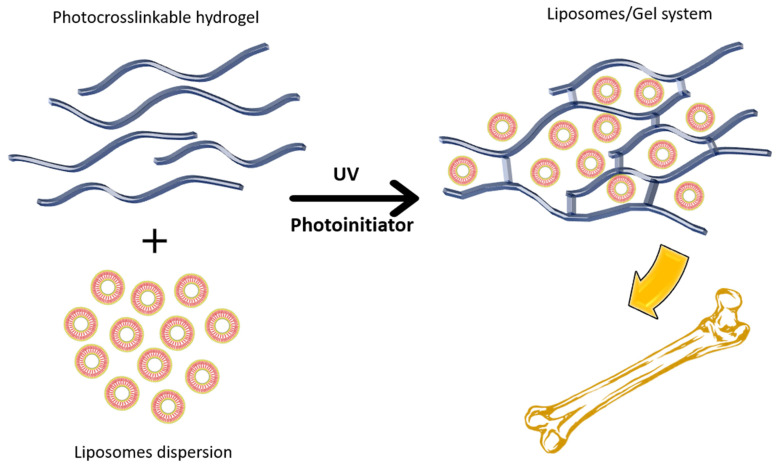
Liposome- hydrogel with enhanced with enhanced residence time over bone.

The immobilization of liposomes using hydrogel has been represented in Figure 6.

The need for a gelification system is supported by a study, in which higher anti-cancer activity has been shown by SLN loaded with resveratrol as compared to resveratrol in the free form. Further, the cell proliferation of the breast cancer cell line(MDA-MB-231) has been arrested in a dose-dependent manner. The resveratrol -SLN treatment also reduced the level of Bcl-2. However, the resveratrol-SLN presented satisfactory results based on the in vitro model only. In the in vivo model, they cannot be applied long because of less residence time at the site of application, especially in bones [107]. 

Therefore, gelatin methacrylate-based hydrogel has been selected to encapsulate SLN containing resveratrol to promote osteogenic differentiation of bone marrow mesenchymal stem cells and bone formation. The SLN managed sustained release of resveratrol with remarkable biocompatibility. Interestingly, this system has been implanted to rat cranial defects, which were completely healed within 8 weeks after the surgery. Therefore, enhanced retention time was obtained by loading the lipid nanoparticulate system into hydrogel [104].

Aside from this, co-encapsulation of anticancer moiety and bisphosphonates into gellified systems also might be an effective approach. It decreases the possibility of further drug dilution and facilitates the drug entrapping also into the outer phase of gel along with minimum drug loss [108]. The aforementioned strategy has been followed, wherein injectable thermo-sensitive poly (L-valine) hydrogel containing methotrexate and alendronate was employed in osteosarcoma progression, which managed sustainable release at the tumor site. More precisely, this developed system has reduced bone destruction and lung metastasis due to osteosarcoma (reduction in bone density, hence weakness) [108,109]. 

Similarly, co-incorporation of oxaliplatin and alendronate into poly(L-valine) hydrogel has achieved gradual immunogenic tumor cell death without causing toxicity to other organs of the mouse osteosarcoma model. The in vivo safety and reliability of the gel is evidence of clinical success in the near future [110]. However, some complications in preparation and applications are also associated with the hydrogels. Therefore, the specific site of application and its clinical effectiveness still need to be explored [109]. 

The SLN are also explored by various researchers worldwide to encapsulate bisphosphonates and have emerged as a suitable carrier to treat osteoporosis. A study focusing on the fabrication of injectable SLN with thermosensitive sol–gel properties is an example of in situ gellified systems in which the low oral bioavailability issue associated with alendronate sodium has been addressed and solved. For this, the high-pressure homogenization technique has been exploited to fabricate SLN (with 68–70% encapsulation efficiency) using Glyceryl monostearate and Tween 80 as a lipid and surfactant, respectively, further subjected to gel formation using Pluronic F-68 as a polymer via the cold method. The SLN gel, having alendronate entrapped, has undergone release kinetic assessment and revealed burst release because of the presence of the drug in polymeric matrix followed by prolonged release up to 94% in the time frame of 102 h. Moreover, the SLN have shown good biocompatibility; no morphological change was spotted at the site of injection [108]. 

Natural and synthetic hydrogels are available along with excellent mechanical features in terms of strength and flexibility [111,112]. The natural gels are not only poor in mechanical strength but also do not allow a wide range of selection of drugs and their burst release. Moreover, the poor mechanical strength of gel further leads to low manufacturability, interfacial separation of the hydrogel, and the entrapped solid always remains a great challenge [113,114]. Recently, a study has been conducted, focusing on enhancing the mechanical strength of liposomal hydrogel via hydrogen bonding between the phospholipid layer of liposomes and the polymeric chain of the gel to achieve precisely controlled drug release. Deferoxamine (hydrophilic drug), paclitaxel (lipophilic drug)), bovine serum albumin (bioactive macromolecule) have been loaded separately to the liposomes prepared by a reverse evaporation method. All the developed systems have been subjected to gellification with a photo-cross linkable gelatin derivative to maintain higher mechanical strength [115]. In this multiple drug delivery model, deferoxamine has shown early release while bovine serum albumin and paclitaxel have shown mid-term and long-term, respectively. Enhanced osteogenic and angiogenesis differentiation has been observed in MC3T3-E1 and HUVECs cells. Lastly, bone formation and tissue construction processes have been accelerated in distal femoral defects of Sprague-Dawley rats [113].

The drug molecule entrapped in gels upon implantation in an organism could release, mix, and be diluted with body fluids. This could be a very serious concern specifically in the anti-cancer drugs, where they can also move to normal cells from cancerous cells and give rise to toxicity [116]. For this, adhesion hydrogel has been selected to enhance liposome adhesion to the bone. For this purpose, mercaptan is a very good candidate, having the ability to form disulfide bonds to the cysteine remainder of mucus in the tissue [117]. However, this bonding is for a short time, and hence, is reversible in nature and does not allow persistent adhesion of the liposomes. Another choice as a mucoadhesive agent is chitosan but it deposits on the surface of liposomes very weakly. On the other hand, the hydrophilic nature of the chitosan derivatives hinders hydrogel formation. To solve the above-highlighted difficulties, octadecylamine as a film-forming material has been projected and can be associated tightly along with the liposomes, and preventing its mixing with body fluid will facilitate long-term adhesion of the liposomes. These adhesive liposomes were further loaded into PEG-hydrogel and injected into the osteoporotic fracture, where the liposomal gel was adhered to and released its active molecule successfully, ultimately promoting bone regeneration without affecting cell proliferation. Comparatively to ordinary gel, the adhesive liposomal gel has shown better remodeling in rat models. Moreover, the system has shown remarkable injectability, antibacterial, and self-healing properties to avoid the risk of infection after surgery [116].

To treat fractures, bone tumors, and osteoporosis, autotransplantation is a globally accepted strategy. However, it needs at least two surgeries. Additionally, the allograft is another way but may face immunological dismissal. A biomimetic approach using exosomes can be employed [118,119]. Herein, human umbilical cord mesenchymal stem cell-derived exosomes have been used. Coralline hydroxyapatite/silk fibroin/glycol chitosan/difunctionalized polyethylene glycol self-healing gel has shown excellent bone repair performance. Consequently, exosomes and hydrogel were successfully established with synergism in the process of bone collagen deposition, maturation, and angiogenesis [79]. 

## 3. Composition of Lipid-Based Systems in Bone Metastasis and Bone Regeneration

An ideal drug delivery system should unload the encapsulated therapeutic moiety at a specific site along with the desired concentration. There are various contributing factors responsible for the overall performance of the transporter, including choice of drug, composition, selection, and optimization of method of preparation, target site [120]. Several studies have emphasized the comparisons of different lipid matrices and their overall effect on the performance of lipid nanoparticles [121]. Further, the composition also depends upon the site of targeting. For instance, to infiltrate a negatively charged cell membrane and deliver cargo in the cytoplasm deeply, the carrier must have a positive surface charge. Otherwise, neutral and anionic charges cannot perform this task [17]. As per the previous discussion, the selection of targeting moiety plays a key role, especially in cancer treatment strategies. Attachment of different targeting agents namely bisphosphonates, proteins/genes, and growth factors, make the system more efficient. Some examples supporting the above statement have been mentioned in Table 2.

The route of administration is a crucial parameter when designing a drug delivery system. For instance, alendronate is responsible for gastrointestinal intolerance evidenced by ulceration or local irritation, intravenous administration leads to nephrotoxicity, subcutaneous and intramuscular intake cause local irritation and tissue damage are main disquiets. Notwithstanding, pulmonary delivery is always assumed to be a safer or better alternative to the injectable route. Considering this, Compritol 888 ATO-SLN comprising alendronate were produced for pulmonary delivery were reported with minor toxicity assessed on A549 cell line via cytotoxicity analysis as a result of no use of any organic solvent in the production procedure [122]. Therefore, the composition highly depends upon the proposed route of administration. The optimization of the concentration of the excipients and process variables can be done to obtain the best composition ratios [123,124,125,126].

**Table 2:** Different lipid compositions and targeting moieties exploited in bone targeting.

Carrier	Drug	Composition	Targeting Moiety	Outcome	Ref.
Liposomes	Paclitaxel	Soybean phospholipids	Glutamic oligopeptides-RGD peptide	High hydroxyapatite binding efficiency, improved cytotoxicity	[127]
	Doxorubicin	Hydrogenated soy phosphatidylcholine, cholesterol and DSPE-mPEG2000	Aspartate and folate	relieve pain and improve survival in a mice model	[128]
	Doxorubicin	Distearoylphosphotidylcholine, cholesterol	Thiol-bisphosphonate	A good candidate in bone regeneration with higher retention	[129]
Lipid Nanoparticles	Glucocorticoid prednisolone	Glyceryl monostearate, dimethyldioctadecylammonium bromide, cholesterol	Hyaluronic acid	Reduced joint swelling, bone erosion, and levels of cytokines in serum	[130]
	Simvastatin	monostearin, polyethylene glycol monostearate, oleic acid	Aspartic oligopeptide	Induced osteoblast differentiation, biocompatible with MC3T3-E1 cells	[27]
	Bone morphogenetic protein-9 gene	DOPE (1,2-dioleoyl-sn-glycero-3- phosphoethanolamine), mPEG2000-DSPE (1,2-distearoyl-sn-glycero-3-phosphoethanolaminemethoxypolyethyleneglycol 2000), hydrogenated soy phosphatidylcholine, and cholesterol	Bone-homing peptide	Effective in vitro and in vivo gene delivery, no toxicity	[131]
	siRNA	Dilinoleylmethyl-4-dimethylaminobutyrate, distearoylphosphatidylcholine, cholesterol, and polyethylene glycol-dimyristol glycerol	N/A	Prolonged knockdown, accumulation of osteocytes	[132]

## 4. Characterization of Lipid-Based Nano-Sized Cargos

The physicochemical characterization is the foremost most crucial requirement which can give the idea about the success of further in vivo and in vitro experiments. Therefore, these characterization parameters will be responsible for the efficiency of the final product. The basic physical characterization can be done by considering average diameter, PDI, zeta potential, and internal structural organization by SAXS, whilst the chemical characterization generally includes % entrapment efficiency, drug release profile, chemical stability of embedded compound over the time of storage [101]. The diameter affects the overall fate of the NP and should be around 200–400 nm depending upon the target organ of delivery, and polydispersity (PDI) should be below 0.3 to support the fact that dispersion is homogeneous with good quality. The diameter and polydispersity can be evaluated by Dynamic Light Scattering and The Sedimentation Field-Flow Fractionation. Further, Zeta potential (measurement technique is based on electrophoresis) is useful to measure the surface charge on the nanomaterial responsible for their better colloidal stability [121]. The use of cationic and anionic surfactants can shift the zeta potential values from positive to negative, respectively [17]. Some physicochemical parameters of the lipid-based transporters have been discussed in Table 3.

Further, the stability of the drug or cargo in the final lipid nanoformulations is also a very important aspect. However, storage of lipid Nano formulation is a great challenge for its scaling up. In this regard, storage temperature plays a vital role and should be determined. Plenty of attempts have been made in order to understand the role of the storage temperature on the overall stability and efficiency of the system. For this, different temperature conditions were applied to different formulations. Two very important approaches should be considered here. Firstly, the objective to employ a lipid nanocarrier is to protect the cargo from degradation over a longer time. One study related to caffeic acid (well-known poly phenol-based anti-oxidant) revealed that the caffeic acid in the aqueous solution is completely unstable at temperatures ranging from 22 °C, 4 °C, or 40 °C degraded in 30 days completely. Whereas, on encapsulation into ethosomes, more than 80% of the molecule was stable even after 6 months [33]. Secondly, the cubosomes meant for transdermal delivery have been prepared to load different anti-oxidant molecules and the final formulation was stored at different temperatures and evaluated by change in diameter over the time of six months. Interestingly, it has been found that they were stable at room temperature. The storage of products at room temperature will reduce the overall cost of the products [139].

Concerning production and stability, SLN are very attractive with low cost. They can be highly stable for three years at least, depending upon the type of lipid, concentration of surfactant, and temperature conditions during production. Further, the rate of polymorphic transition depends upon the chain length (gradual crystallization with longer length) of the triglycerides [140]. In some cases, it is not possible to store at room temperature because of the type of drug, then these products can be subjected to lyophilization and spray drying [141].

## 5. Challenges and Future Prospectives

Even though lipid-based nanoformulations in drug delivery provide a wide range of applications and have gained great success at laboratory scales, some obstructions still exist that need to be addressed and solved. The lipid nanoparticles are still far away from clinical evaluation, evidenced by the recent publications, which are mostly focused only on technological development and thus, are not disease-oriented [142]. A very limited number of lipid nano-products that achieved clinical success can be seen clearly from the last 50 years of research. Both the factors, either the complexity of the manufacturing process, lack of robustness in the characterization techniques, or strict government regulations are responsible for that. Further, the surface modification, ligand conjugation, and coating turn the lipid formulation highly complex, which creates problems in reproducibility [101,143]. More and more characterization techniques need to be added due to complex structural organization. Therefore, the number of process variables will also be increased. Hence, conducting clinical trials with these numerous process variables is much harder in the lipid nanomaterial compared to conventional drug transporters.

Exosomes are naturally derived vesicles and do not have production-related hurdles. However, they are facing difficulty in the lack of a versatile method of their isolation and collection, specific mechanisms of action, quantification, and characterization in body fluids, stability of engineered exosomes [144]. Further, some release profiles from exosome integrated scaffolds reveal that assimilating exosomes to scaffolds via physical adsorption is not enough to retain exosomes for a longer time, especially in the curing of bone defects. Lastly, some other methodologies need to be developed [145].

In the end, the high production cost can also not be avoided among the hurdles in the scale-up process. Production costs can be reduced by choosing a cost-effective production method of fabrication. Further, the more economic carrier can be selected, such as niosomes is an alternative to the liposomes, can be prepared cheaply, and have commendable performance. Similarly, SLN can also cut the production cost by avoiding the use of organic solvents and sophisticated production procedures, unlike liposomes. They can be produced very easily by high-pressure homogenization and the very much biocompatible. They can also embed both hydrophilic and hydrophobic active molecules. The combination of polymer and lipid for dual functioning is possible. They also give the possibility to modify the surface for gene delivery [120,141,146].

Recently, a technique has been proposed to prepare liposomes by avoiding the use of organic solvent, named as supercritical carbon dioxide technique. It is an inexpensive, inert, harmless, fire-resistant, and environmentally friendly approach, in which atomized water droplets are used to coat phospholipid vesicles under high diffusion of carbon dioxide. In this, size and shape can be controlled precisely [147,148]. Cholecalciferol-loaded nanoliposomes were prepared with this approach and 66.7 to 88.9% encapsulation efficiency has been achieved [149]. This process is also useful in the production of the niosomes containing theophylline with 85% encapsulation. The optimization of surfactant and lipid is also possible, which extends the release by five-fold [150].

## 6. Conclusions

In this review, the design, production, and in vivo/in vitro investigations of lipid-based nanomaterial in bone targeting have been discussed in detail. It is highlighted that these carriers can perform drug/gene/protein delivery efficiently. Moreover, various agents, namely bisphosphonates and calcium phosphate with high bone affinity can be employed as lipid carriers to establish the synergism. Apart from these transporters composed of synthetic lipid or manufactured artificially, exosomes from a natural origin have been introduced. Furthermore, to make these systems more useful at the clinical level with high residence time, the concept of hydrogel has been highlighted. Light is also shed on the approaches to make the hydrogel mechanically stronger. The importance of the parameters for physicochemical characterization has been discussed. Lastly, the stability of the final product at different temperatures, the use of alternative techniques where the use of organic solvent and sophisticated technology can be avoided to make the nanomedicine products cost-effective was also discussed. Taken all together, it is most important to work on these strategies to make them suitable for clinical trials and also to scale up with maximum cut-off in production cost.

## Figures and Tables

**Table 1:** Issues related to drugs in bone targeting.

Drug	Issue	Formulation	Outcome	Ref.
Metvan	Rapid oxidation, interference with blood components	Nanostructured Lipid Carriers	Quantitative encapsulation efficiency, sustained-release within 48 h, high cytotoxic effects	[25]
Icariin	Low water-solubility, susceptible to first-pass metabolism, and low bioavailability	Liposomes	Amplified the mechanical strength of femoral midshaft, triggered bone turnover/remodeling	[26]
Simvastatin	Deterioration at a physiological pH, low water solubility, low bioavailability, high toxicity	Lipid nanoparticles	Higher encapsulation efficiency with a sustained release of 70% within 50 h, reduction in cytotoxicity	[27]
Doxycycline	Degradation in the anhydrous environment, poor bone penetration	Lipid- Polymer hybrid system	Zero-order release rate up to one month, eradicate bacterial bone infections	[28]
Edelfosine	Poor oral bioavailability, dose-dependent hemolysis	Lipid nanoparticles	Shows immediate cytotoxicity to human osteosarcoma cells, negligible tumor growth with declining of tumor volume by five-fold	[29]
TNF-α small interfering RNA	Short half-life, deprived extravasation from blood vessels to target cells, low cellular uptake	PEGylated solid-lipid nanoparticles	Encapsulation efficiency more than 90%, precise targeting to inflamed sites in a mouse model, declined bone loss,	[30]

**Table 3:** Characterization parameters of lipid nanosystem developed for bone metastasis.

Formulation	Cargo	Avg. Diameter (nm)	PDI	Zeta Potential (mV)	%EE	Ref.
Liposomes	Sodium-alendronate	185.2 ± 22	<0.3	−27.4 ± 1	N/A	[133]
		160 ± 24	<0.1	–29.2 ± 1.9	30 ± 5	[37]
		298 ± 3.5	0.07 ± 0.2	−39 ± 2.19	78.5	[134]
Lipid nanoparticles	Edelfosine	124 ± 12	0.16 ± 0.01	−14.5	N/A	[29]
	Docetaxel	128 ± 2.2	0.153 ± 0.02.	− 15 ± 0.5	86 ± 2.4	[135]
	Berbamine	75	<0.3	−16	87	[136]
	Mitoxantrone	230 ± 17	0.16 ± 0.01	−3 ± 1	93 ± 6	[137]
	Tamoxifen	277.4 ± 1.26	0.298 ± 0.05	−40.5 ± 1.61	N/A	[138]

## Data Availability

Not Applicable.
